# Prehospital Diagnosis of Shortness of Breath Caused by Profound Metformin-Associated Metabolic Acidosis

**DOI:** 10.3390/healthcare9010074

**Published:** 2021-01-14

**Authors:** Pietro Elias Fubini, Laurent Suppan

**Affiliations:** 1 Division of Intensive Care Medicine, Department of Anaesthesiology, Clinical Pharmacology, Intensive Care and Emergency Medicine, Geneva University Hospitals and Faculty of Medicine University of Geneva, CH-1211 Geneva, Switzerland; 2 Division of Emergency Medicine, Department of Anaesthesiology, Clinical Pharmacology, Intensive Care and Emergency Medicine, Geneva University Hospitals and Faculty of Medicine University of Geneva, CH-1211 Geneva, Switzerland; laurent.suppan@hcuge.ch

**Keywords:** prehospital, arterial blood gas, metformin poisoning, metabolic acidosis, respiratory distress

## Abstract

Shortness of breath is a common complaint among patients in emergency medicine. While most common causes are usually promptly identified, less frequent aetiologies might be challenging to diagnose, especially in the pre-hospital setting. We report a case of prehospital dyspnoea initially ascribed to pulmonary oedema which turned out to be the result of profound metformin-associated metabolic acidosis. This diagnosis was already made during the prehospital phase by virtue of arterial blood gas measurement. Pre-hospital measurement of arterial blood gases is therefore feasible and can improve diagnostic accuracy in the field, thus avoiding unnecessary delay and potential harm to the patient before initiating the appropriate therapeutic actions.

## 1. Introduction

Shortness of breath (SOB) is one of the most common complaints among patients in prehospital emergency medicine [1]. Most common causes of shortness of breath (pulmonary oedema, pneumonia, chronic obstructive pulmonary disease exacerbation, pulmonary embolism, pneumothorax, etc.) are usually identified rather easily, although less common aetiologies might be overlooked and therefore misdiagnosed if clinicians fail to consider them. Furthermore, even a high clinical suspicion does not equal a definitive diagnosis, which might require para-clinical confirmation through specific investigations such as arterial blood gas (ABG) analysis. We report the case of a patient complaining of SOB that was initially imputed to pulmonary oedema. However, an ABG analysis performed in the field allowed the prehospital emergency physician to identify a profound metformin-associated metabolic acidosis as the actual cause of the patient’s dyspnoea.

## 2. Case Description

The emergency medical call centre (EMCC) dispatched an advanced life support ambulance to take care of a 71-year-old woman who had called complaining about SOB. Upon arrival, prehospital providers noted shallow breathing with a respiratory rate of over 35 breaths per minute. Lung fields were clear to auscultation and the patient showed no sign of cyanosis. There was no haemodynamic compromise. The Glasgow Coma Scale was 15 with no focal deficit and the patient was normothermic. Given the respiratory distress, paramedics initiated oxygen therapy using a high-flow non-rebreather mask. Suspecting acute heart failure (AHF), they asked the EMCC to dispatch a medical mobile unit called ‘Service Mobile d’Urgence et de Réanimation’ (SMUR). These units are staffed by an advanced paramedic and a physician and can be dispatched to assist an ambulance in case of life-threatening emergencies [2]. These SMURs are the only prehospital units equipped with non-invasive ventilation (NIV) devices and portable blood gas analysers (iStat, Abbott Point of Care Inc., Princeton, NJ, USA) and are therefore often called upon to enhance the management of patients in acute respiratory distress or failure. While awaiting the arrival of the SMUR, the paramedics continued their evaluation and discovered that the patient was under metformin for diabetes and diuretics for hypertension, and that she was on day 3 of antibiotic treatment for urinary tract infection. She did not use cigarettes, alcohol, or illicit drugs. The patient was found to be hypoglycaemic at 2.3 mmol/L and received intravenous glucose accordingly. An ABG analysis performed by the SMUR physician shortly after arriving on site showed severe lactic acidosis (FiO_2_ 65%: pH 6.845, PaCO_2_ 1.95 kPa, PaO_2_ 27.7 kPa, HCO_3_^−^—2.5 mmol/L, lactate 17.0 mmol/L).

While AHF was suspected at first, the profound metabolic acidosis revealed by the ABG analysis was not compatible with the clinical picture. Indeed, the patient was not in cardiogenic shock, and a diagnosis of lactic acidosis secondary to metformin intoxication was thereafter suspected. Intravenous hydration was started and bicarbonates were administered. Since the patient showed signs of respiratory distress and exhaustion, NIV was initiated.

In-hospital investigations confirmed severe lactic acidosis and acute kidney injury KDIGO stage 3 [3], with a creatinine of 788 µmol/L which yielded an estimated glomerular filtration rate of 5 mL/min/m^2^ according to the Chronic Kidney Disease Epidemiology Collaboration (CKD-EPI) equation. This kidney injury was secondary to the pre-renal disease on severe dehydration (diuretics, decreased drinking inputs, etc.) and sepsis, with consequent acute tubular necrosis. The patient showed no sign of hepatic impairment nor hypoxaemia. There was no hint that the patient’s illness could have been secondary to suicidal or accidental biguanide ingestion. The diagnosis of profound metformin-associated metabolic acidosis secondary to renal failure was definitively established at this stage. Despite aggressive hydration and bicarbonates infusion, the patient remained oligo-anuric with severe metabolic acidosis and showed signs of respiratory exhaustion finally requiring endotracheal intubation. She was admitted to the intensive care unit (ICU) for haemodynamic and respiratory management. The decision to start haemodialysis was finally made basing on the severity of acidosis and lactate level, coexisting renal failure and failure to improve with bicarbonate therapy [4]. The patient improved rapidly under this treatment and was extubated on the next day after pH normalisation and lactate clearance. She was transferred to the internal medicine ward after 48 h and finally discharged from the hospital 10 days later, after full renal recovery.

## 3. Discussion

SOB is a major complaint among pre-hospital and ED patients, accounting for over 3.9 million visits in 2017 in the United States [5]. Most of these visits involve patients over 65 years old. Generally, cases of SOB are readily identified and can be ascribed to frequent aetiologies, i.e., heart failure (43%), community-acquired pneumonia (35%), acute exacerbation of chronic respiratory disease (32%), pulmonary embolism (18%), and acute asthma (3%). Two coexisting causes of SOB are found in as many as 47% of patients [6]. Nevertheless, clinicians must be aware that other less frequent aetiologies of SOB should be considered (salicylate intoxication, carbon monoxide poisoning, toxin-related metabolic acidosis, diabetic ketoacidosis, sepsis, anaemia, etc.). They should also acknowledge that diagnosing infrequent causes of SOB might be challenging, especially in the pre-hospital setting, where resources are limited and most investigation tools unavailable. Inappropriate dismissal of infrequent causes of SOB might lead to delayed diagnosis and treatment. Such delays might prove harmful, most particularly in old and frail patients.

Our case exemplifies how pre-hospital ABG measurement can easily improve diagnostic accuracy in such cases. In our prehospital system and in many European countries, trained physicians can be dispatched by EMCCs to provide medical care in the field [7], and can therefore draw and analyze ABG. However, even though recent and compact point-of-care testing devices allow for quick and reliable ABG analysis in the field [8], most emergency medical systems still lack such tools in their standard equipment. Barriers to the implementation of ABG analysis devices in the field have scarcely been described, but might include the absence of prehospital emergency physicians to interpret the results and the fear of unnecessarily delaying transport. Prehospital systems unable to dispatch emergency physicians in the field could however develop specific procedures to allow the use of and interpretation of ABG. Paramedics could be trained to draw arterial blood, protocols could be created to help paramedics determine cases in which ABG analysis could be of use, and distance interpretation by medical supervisors could be considered. Similar procedures have been successfully developed regarding electrocardiographic interpretation [9,10]. In the absence of distance medical supervision, regular algorithms or even electronic apps could be developed to facilitate the interpretation of ABG results [11,12]. Unnecessarily delaying transport could be avoided by limiting and clearly identifying the indications to ABG analysis, and by limiting the number of attempts and the time allowed to draw arterial blood. Moreover, other procedures can be performed while blood is drawn and analyzed, thereby further limiting potential delays. In our system, physicians are allowed only one attempt in a maximum of 60 s.

Absolute contraindications to ABG sampling can usually be clinically identified easily (local infection, thrombosis or distorted anatomy, severe peripheral vascular disease, abnormal Allen’s test, etc.), and serious complications (infection, arterial occlusion or embolism, nerve injury, pseudo-aneurysm or vessel laceration, etc.) are rare. Minor complications (local pain, minor bleeding or hematoma, vasovagal response, etc.) can be easily managed and anticoagulants or antiplatelet agents do not represent absolute contraindications but often require only increased vascular pressure. Although venous sampling could lead to misdiagnosis, providers can be trained to recognize such samples.

In this particular case, onsite ABG analysis allowed the physician to suspect metformin-associated metabolic acidosis and start the appropriate treatment even in the pre-hospital setting. While previous studies have failed to show a general positive effect of prehospital ABG on diagnostic accuracy, it can be of significant benefit in specific situations [13]. Moreover, ABG has been shown to improve treatment quality in critically ill patients [13]. In our system, prehospital physicians notify the Emergency Department (ED) team through the EMCC and can activate specific medical support before arrival. Consultants are thus notified before the patient’s arrival in the ED, thereby accelerating the initiation of appropriate and definitive care. In our institution, the decision to initiate haemodialysis is made either by nephrologists or by ICU physicians. In this particular case, the prehospital physician asked for the ICU consultant to be present upon arrival in the ED based on the suspected diagnosis. The patient was therefore quickly assessed by a multidisciplinary team, and therapeutic decisions were rapidly made. All further necessary steps (intubation, ICU admission, dialysis catheter insertion, haemodialysis initiation) were quickly initiated, and the time spent in the ED was therefore limited to a minimum.

Metformin-associated metabolic acidosis is a rare but well-known complication of metformin treatment. Its incidence was calculated to be 4.3 cases per 100,000 patient-years [14]. When it develops, mortality rates as high as 48% have been reported [15,16]. Clinically significant metformin intoxication and lactic acid accumulation almost always occur in the presence of comorbid conditions, such as impaired kidney function. In our case, the patient indeed presented a KDIGO stage 3 acute kidney injury due to pre-renal disease consecutive to severe dehydration and sepsis. Prompt identification and treatment are mandatory in such cases, but diagnosis might be particularly difficult because of aspecific presentation. Moreover, acidaemia can be profound even though the patient does not seem to be critically ill.

Although the main cause of the lactic acidosis found on ABG analysis was rapidly identified in this case, other potential aetiologies should be always considered. These include severe sepsis, shock, anemia, seizures, hypoxemia, carbon monoxide or cyanide poisoning, ethylene glycol or salicylates intoxication, alcoholism and liver disease, beta-agonist medication or profound thiamine deficit [17]. In our situation, these aetiologies were rapidly excluded by clinical appreciation and laboratory analysis.

The role of bicarbonates infusion is still debated in metformin-associated metabolic acidosis as in other causes of anion gap metabolic acidosis [18]. In our case, this treatment was administered given the profound acidaemia and the signs of respiratory exhaustion, with the aim of avoiding endotracheal intubation. As the patient did not improve under supportive care and bicarbonate infusion, the decision to employ extracorporeal removal was swiftly made and, by virtue of a rapid diagnostic path, haemodialysis was started approximately 6 hours after the first medical contact. Haemodialysis has been shown to be effective in cases of metformin-associated metabolic acidoses [19] and is recommended in patients showing signs of severe poisoning. These signs include a lactate concentration higher than 20 mmol/L, a pH ≤ 7.0, or failure to improve with standard treatment within a few hours [4] as in our case.

## 4. Conclusions

This case report shows that prehospital ABG measurement is feasible and can improve diagnostic accuracy in the field. In this particular case, ABG analysis enabled the prehospital physician to make the correct diagnosis in a timely manner, thus allowing the appropriate treatment to be initiated before hospital admission. Unnecessary delay and potential harm to the patient were thereby avoided.

## Data Availability

Not applicable.
